# A rapid cosmic-ray increase in BC 3372–3371 from ancient buried tree rings in China

**DOI:** 10.1038/s41467-017-01698-8

**Published:** 2017-11-14

**Authors:** F. Y. Wang, H. Yu, Y. C. Zou, Z. G. Dai, K. S. Cheng

**Affiliations:** 1 School of Astronomy and Space Science, Nanjing University, Nanjing, 210023 China; 2 Key Laboratory of Modern Astronomy and Astrophysics (Nanjing University), Ministry of Education, Nanjing, 210023 China; 3 School of Physics, Huazhong University of Science and Technology, Wuhan, 430074 China; 4 Department of Physics, University of Hong Kong, Hong Kong, China

## Abstract

Cosmic rays interact with the Earth’s atmosphere to produce ^14^C, which can be absorbed by trees. Therefore, rapid increases of ^14^C in tree rings can be used to probe previous cosmic-ray events. By this method, three ^14^C rapidly increasing events have been found. Plausible causes of these events include large solar proton events, supernovae, or short gamma-ray bursts. However, due to the lack of measurements of ^14^C by year, the occurrence frequency of such ^14^C rapidly increasing events is poorly known. In addition, rapid increases may be hidden in the IntCal13 data with five-year resolution. Here we report the result of ^14^C measurements using an ancient buried tree during the period between bc 3388 and 3358. We found a rapid increase of about 9‰ in the ^14^C content from bc 3372 to bc 3371. We suggest that this event could originate from a large solar proton event.

## Abstract (web summary)

^14^C can be absorbed by trees as a result of the interaction of cosmic rays produced by high-energy phenomena with the Earth’s atmosphere. Here, the authors observe a rapid increase of ^14^C in an ancient buried tree from BC 3372 to BC 3371, and suggest that it could originate from a large solar proton event.

## Introduction

The cosmogenic nuclide ^14^C is produced in the Earth’s atmosphere through neutrons captured by nitrogen nuclei. These neutrons are generated by cosmic rays interacting with the atmosphere. Through the global carbon cycle, some of ^14^CO_2_ produced in the atmosphere can be retained in annual tree rings^1–3^. Therefore, ^14^C concentrations in annual tree rings indicate the intensity of cosmic rays. The flux of cosmic rays reaching the Earth is modulated by time variations of geomagnetic and heliomagnetic fields. The international radiocarbon calibration curve IntCal13 contains tree-ring records of ^14^C data with a typical five-year resolution extending to 13,900 years before the present^4^. The IntCal13 curve shows variations due to solar and geomagnetic activities on a decadal to millennial time scale. However, there are only a few annual ^14^C data measured from tree rings. So some rapid ^14^C increases caused by cosmic-ray events may be hidden in the IntCal13.

Interestingly, a large amount of cosmic rays can be generated on a short time scale by high-energy phenomena, such as supernovae (SNe)^5^ and large solar proton events (SPEs)^6–8^. Meanwhile, the energy deposited in gamma-rays of SNe^1^ and short gamma-ray bursts (GRBs)^9^ can also cause a rapid ^14^C increase. Accordingly, rapid increases of ^14^C content in tree rings are valuable tools to explore high-energy phenomena occurred in ancient times.

Recently, two events of rapidly increasing ^14^C content occurring in ad 774–775 and ad 993–994 were found using Japanese trees^6, 10^. More recently, a sharp increase of ^14^C content about 10‰ within about 3 years was found at bc 660^11^. The ad 774–775 event was confirmed by other tree samples in different places^8, 12, 13^, indicating that this event is worldwide. Several possible causes for these events have been proposed. Supernova remnants occurring at ad 774, ad 993, and bc 660 have not been observed and historical record of SNe has not been found. So, the three reported events are unlikely caused by SNe^11, 14, 15^. The other possible explanations of the three events are short GRBs^14, 16^ or large SPEs^6, 8, 11, 12^. The ^10^Be measurements in ice cores from Antarctica, Greenland, and Arctic also show a spike around ad 775^17, 18^, which indicates that a large SPE is the most likely explanation. But whether short GRBs could generate substantial increase in ^10^Be remains unclear^14, 16^. Meanwhile, the local event rate of short GRBs is much smaller than the rate of ^14^C increase events. Other attempts have been made to search for rapid ^14^C increases. For example, the ^14^C content has been measured in bristlecone pine tree-ring samples in bc 2479–2455, bc 4055–4031, bc 4465–4441, and bc 4689–4681^19^. But no large ^14^C increases during these periods are found. It is possible that there were other rapid ^14^C increases in the past, undetected due to the lack of annual ^14^C measurements. In order to find more rapid increases in ^14^C data at annual resolution, we select the periods during which the ^14^C value increases significantly in the IntCal13 data. There are two intervals where the increase rate is >0.6‰ per year between bc 3380 and bc 3370. It is possible that larger annual changes hide in the averaged five-year data.

Here, we report the measurement of ^14^C content for an ancient buried tree in China during the period bc 3388–3358 to search ^14^C increase events, and find a rapid increase from bc 3372 to bc 3371. Considering the occurrence rate of the rapid ^14^C increase events, the ^14^C event could originate from a large SPE.

## Results

### Wood sample

We use a Chinese wingnut (*Pterocarya stenoptera*) tree, which was found in the city of Yichang, Hubei Province (30°31′N, 111°35′E), China. The sample of Chinese wingnut is housed in the Yichang museum. The tree is shown in Supplementary Fig. 1. This type of wood was formed in the following way. Living trees were buried in river bed or low-lying place by an earthquake, floods, or debris flows. Then, after thousands of years of carbonization process, this type of wood would be formed. The carbonization process is the early stage of the coalification process^20^, which can be taken as the slow hydrothermal carbonization process^21^, and the annual rings are preserved. The buried wood was first introduced to the West by Ernest Henry Wilson in 1913^22^. It is regarded as priceless raw material for carving. It also has substantial artistic and scientific research values, such as revealing forest information, for studying paleoclimate, and speculating on natural disasters.

Unlike very aging trees, the ancient buried woods are relatively common in nature. The period of buried woods spans a long era back. On the other hand, unlike coal, the buried woods still contain the structure of the trees. Therefore, the ancient buried woods are good samples of the carbon abundance research on past epochs up to tens of thousand years ago, as well as on other plant archeology. For example, the use of the sample may extend the data of IntCal13.

The tree was dated with tree-ring records using standard dendrochronology. We used the master chronology of tree-ring widths from California (https://www.ncdc.noaa.gov/data-access/paleoclimatology-data). The program dpLR is used to perform the dendrochronology^23^. We found that the correlation value with the master is 0.525. The possible age error of the wood sample is about 2 years. Considering the age error of ^14^C dating is about 20 years, the age from dendrochronology is consistent with that from ^14^C dating. We separated annual rings carefully using a knife. The cellulose samples are prepared by standard chemical cleaning methods. The tree rings are measured using the Accelerator Mass Spectrometry (AMS) method at the Beta Analytic radiocarbon dating laboratory (http://www.radiocarbon.com/). In order to cross-check our results, we also measured another sheet of wood from the same tree (as a different sample) at the Institute of Accelerator Analysis laboratory (IAA) (http://www.iaa-ams.co.jp/indexen.html).

### Measurement data

**Fig. 1:**
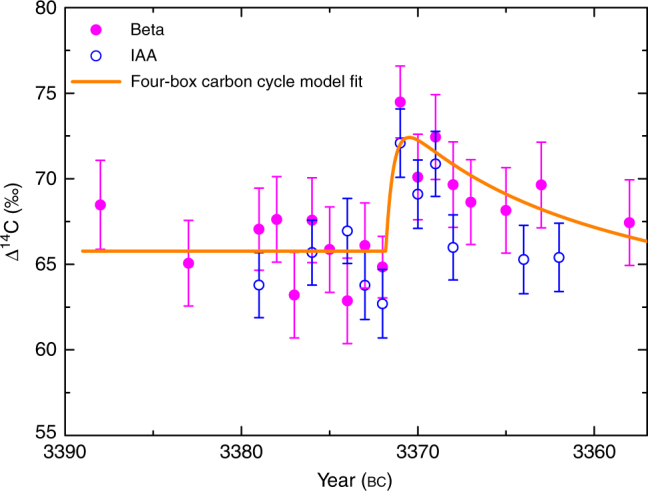
Measured ^14^C content. Measured results of Δ^14^C for the tree rings using the AMS method at the Beta Analytic radiocarbon dating laboratory (filled circles) and the Institute of Accelerator Analysis laboratory (open circles). The typical error of a single measurement is about 2.5‰ for filled circles and 2.0‰ for open circles. In order to obtain a smaller error for this ^14^C increase event, several measurements for bc 3371 and bc 3372 are performed. The solid line is the best fit for filled circles using the four-box carbon cycle model with a net ^14^C production of *Q* = (7.2 ± 1.2) × 10^7^ atoms cm^−2^. Uncertainties (s.d.) are based on error propagation including measurement errors of the fraction of modern carbon *F*

**Fig. 2:**
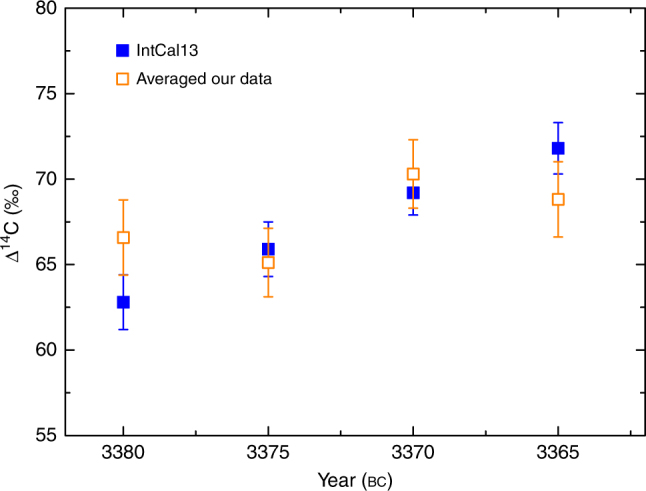
Comparison with IntCal13 data. Comparison of our five-year average of Δ^14^C data measured in the Beta Analytic laboratory (open squares) with the IntCal13 data (filled squares)^4^. They are generally consistent with each other considering the measurement errors. Uncertainties (s.d.) of our data are based on error propagation including measurement errors of the fraction of modern carbon *F*. The errors of filled squares are adopted from IntCal13^4^

**Fig. 3:**
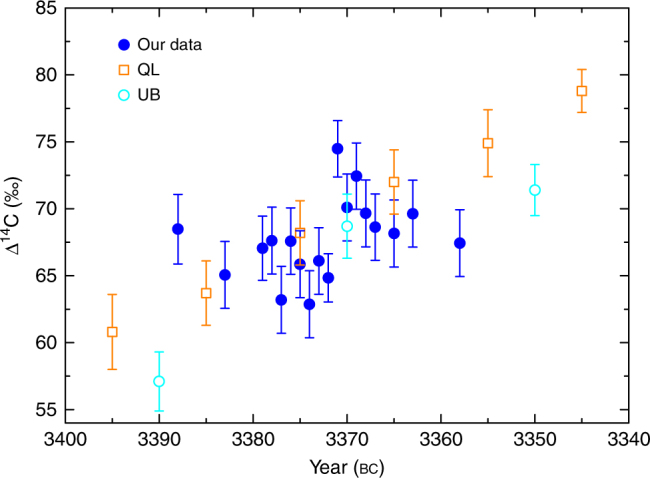
Comparison with IntCal13 original data. Comparison of our data measured at the Beta laboratory (filled dots) with the IntCal13 original data (The UW Quaternary Isotope Laboratory (QL, open squares)^26^, The Queen’s University of Belfast (UB, open dots)^27^). For the three Δ^14^C value measured in the same year, they are consistent with each other within measurement errors. The value of Δ^14^C at bc 3385 from QL is between the nearby two points measured in Beta laboratory. Therefore, our measured results well agree with the IntCal13 original data considering the measurement errors. Uncertainties (s.d.) are based on error propagation including measurement errors of the fraction of modern carbon *F*

In general, AMS measures the fraction of modern carbon *F*, *δ* ^13^C, and Δ^14^C. The definitions of these values can be found in ref. ^24^. First, we measure the ^14^C content between bc 3388 and bc 3358 every 5 years. Then, the yearly measurements of ^14^C content from bc 3379 to bc 3365 are performed, because the ^14^C increase rate is >0.6‰ per year in this period. Figure 1 shows the variation of ^14^C content of the tree rings for the period bc 3388–3358. The filled circles are measured results from the Beta Analytic radiocarbon dating laboratory and the open circles are measured from the Institute of Accelerator Analysis laboratory. The two series of data are consistent with each other (Tables 1 and 2). So the measurement results are reproducible. We found an increase of ^14^C content of about 9.4‰ from bc 3372 to bc 3371. After the increase, a gradual decrease over several years due to the carbon cycle is observed. The significance of this increase with respect to the measurement errors is 5.2*σ*. The profile of this ^14^C event shows a rapid increase within about 1 year followed by a decay due to the carbon cycle, which is similar to the ad 774–775 event. In order to estimate ^14^C production required for this event, we use the four-box carbon cycle model to fit the Δ^14^C data. The four reservoirs are troposphere, stratosphere, biosphere, and surface ocean water^25^. The transfer coefficients of carbon from one reservoir to another used in this work are the same as Miyake et al.^6^. We assume all the ^14^C is injected into troposphere instantaneously. The best-fitting result is shown as the solid line in Fig. 1. The best fit by the weighted least-squares method yields a net ^14^C production of *Q* = (7.2 ± 1.2) × 10^7^ atoms cm^−2^. According to the calculation of Usoskin et al.^8^, the ^14^C production for the ad 774–775 event is (1.3 ± 0.2) × 10^8^ atoms cm^−2^. Therefore, the intensity of this event is about 0.6 times as large as the ad 774–775 event. In order to compare our results with IntCal13^4^, we average the annual data to obtain a series with five-year resolution. The result is shown in Fig. 2. Considering the measurement errors, the two sets of data are consistent with each other. We also compare our data with the original tree-ring data^26, 27^ of IntCal13 in Fig. 3. Interestingly, our measured results well agree with the original data of IntCal13.

**Table 1:** Measured results in the Beta Analytic laboratory

Year (bc)	Δ^14^C (‰)	Error^a^	*δ* ^13^C (‰)
3388	68.47	2.6	−24.9
3383	65.06	2.5	−26.6
3379	67.05	2.4	−26.0
3378	67.62	2.5	−25.6
3377	63.19	2.5	−26.1
3376	67.58	2.48	−25.2
3375	65.86	2.5	−26.3
3374	62.87	2.5	−25.6
3373	66.10	2.49	−24.7
3372	65.03	1.8	−25.4
3371	74.48	2.1	−24.6
3370	70.10	2.5	−25.7
3369	72.44	2.48	−26.4
3368	69.65	2.5	−25.4
3367	68.63	2.48	−25.6
3365	68.15	2.5	−25.5
3363	69.63	2.5	−25.9
3358	67.43	2.5	−24.8

^a^The error of Δ^14^C is calculated from error propagation. In the calculation, the error of the fraction of modern carbon *F* is considered

**Table 2:** Measured results in the Institute of Accelerator Analysis laboratory

Year (bc)	Δ^14^C (‰)	Error^a^	*δ* ^13^C (‰)
3379	63.79	1.9	−26.53
3376	65.68	1.9	−24.77
3374	66.95	1.9	−25.56
3373	63.78	2.0	−27.23
3372	62.70	2.0	−25.88
3371	72.08	2.0	−26.16
3370	69.10	2.0	−24.69
3369	70.87	1.9	−24.97
3368	65.99	1.9	−26.42
3364	65.28	2.0	−25.09
3362	65.40	2.0	−26.04

^a^The error of Δ^14^C is calculated from error propagation. In the calculation, the error of the fraction of modern carbon *F* is considered

## Discussion

The rapid ^14^C increase around bc 3372 must be caused by cosmic high-energy phenomena. The solar cycle cannot produce this large increase. There are several plausible origins for this event.

GRBs are the most powerful electromagnetic explosions in the Universe^28, 29^. According to their duration *T*, they can be divided into long (*T* ≥ 2 s) and short (*T* < 2 s) GRBs. Because the intensity of this event is less than that of ad 774–775 event, the energy of a typical short GRB located at a few kpc can provide necessary energy^14^ for this event. The previous three ^14^C events may not be caused by short GRBs^17^. So, if a short GRB causes this event, it implies that one short GRB explodes in our galaxy about 5,000 years. But the local rate of short GRBs pointed to the Earth is ~10^−5^ yr^−1^ ^7, 15^. So, the short GRB hypothesis is largely ruled out.

SNe are also powerful explosions with high-energy emissions. For a supernova, the ^14^C increase is attributed to both high-energy photons and cosmic rays, but only the high-energy photons would be abrupt. Previous work has shown that a rapid ^14^C increase of 6‰ occurred 3 years after the SN 1006 explosion^1^. However, this result is challenged by a recent study^30^. The ^14^C increase event reported in this paper occurred about 5,300 years ago, at a time from which there is no human historical record. Based on the above calculation, the gamma-ray energy required for this event in the atmosphere is about 10^24^ erg. The typical total energy of a supernova is 10^51^ erg. If a fraction of its total energy, $\eta _\gamma \simeq 1\%$, radiates in gamma-rays, then the supernova must be closer than 326 pc. From the Chandra Catalog of Galactic Supernova Remnants (http://hea-www.cfa.harvard.edu/ChandraSNR/snrcat_gal.html), we found five supernova remnants with distances closer than 400 pc. The possible ages for these five supernova remnants are: *t* = 391 kyr for G006.4 + 04.9^31^, *t* = 4.4 × 10^9^ yr for G014.7 + 09.1^32^, *t* = 3.1 × 10^6^ yr for G047.3–03.8^33^, *t* = 340 kyr for Geminga^34^, and *t* = 2,000–13,000 yr for G266.2–1.2 (Vela Jr.)^35^. Indeed, nearby SNe are rare within the last 300 kyr^36^. Interestingly, the Vela Jr.^37^ locates at hundreds of parsecs and its age is 2,000–13,000 years^35^. From this point of view, a supernova origin of the bc 3372–3371 event appears to be plausible. Unfortunately, *η*_γ_ is much smaller than 1%^38^, so a supernova origin for this event becomes highly implausible.

The most probable origin is a large SPE. Usually, SPEs are associated with solar flares and coronal mass ejections^39^. Due to the uncertainty of the SPE energy spectrum, the estimated fluence of SPE caused the ad 774–775 event varies by as much as two orders of magnitude^8, 40^. Based on more realistic models, Usoskin et al.^8^ found that the ad 774–775 event could be explained by a large SPE, which was about 50 times larger than the SPE in ad 1956^8^. So, the SPE associated the bc 3372–3371 event is about 30 times larger than the SPE in ad 1956. According to calculations by Usoskin et al.^8^, the ad 774–775 event required a, SPE fluence (>30 MeV) of 4.5 × 10^10^ cm^−2^. Because this event is about 0.6 times as large as the ad 774–775 event, this event should correspond to an SPE fluence about 2.7 × 10^10^ cm^−2^, if the same spectrum is assumed. The SPE fluence above 30 MeV of Carrington event is 1.9 × 10^10^ cm^−2^ ^41^, which is about 0.7 times as large as that of bc 3372–3371 event. So, rapid increase of radioisotopes should be detectable around ad 1859. However, neither ^14^C nor ^10^Be peaks were found around ad 1859^10^, which may be attributed to the different spectra of SPEs. If the bc 3372–3371 event is of solar origin, the associated SPE must be extremely powerful. In modern society, such extreme events would damage electronic and power systems^42^, deplete atmospheric ozone^43^, and possibly affect the weather^44^. Based on the four events, the probability of large SPEs is about 7.4 × 10^−4^ yr^−1^. If we assume the energy of an SPE is comparable to that of an X-ray flare, the occurrence frequency of large SPEs is consistent with the frequency of superflares on solar-type stars^45^.

In conclusion, we find a rapid increase of about 9‰ of ^14^C content in buried tree rings from BC 3372 to 3371. Whether this event is worldwide is unknown. Therefore, measuring the ^14^C content of trees in other places around this period is important. The most likely origin of this event is a large SPE. In the future, the measurements of radiocarbon concentration in tree rings are important for studying the large SPEs and cosmic gamma-ray events.

## Methods

### AMS measurement at the Beta Analytic laboratory

Cellulose in tree rings is extracted in the following steps: (1) washing with distilled water; (2) soaking in HCl, NaOH, and HCl solutions; (3) bleaching with hot NaClO_2_ and washing with boiling distilled water. Then, the material was combusted to CO_2_ and converted to graphite using standard procedures. The graphite powders produced are pressed into AMS targets and measured using an AMS system at Beta Analytic radiocarbon dating lab in Miami. The ^14^C/^13^C ratio of the sample is compared to known standards (OxalicI and II, National Institute of Standards and Technology standards SRM 4990B and 4990C, respectively), and the result corrected to the measured value of *δ* ^14^C made off line on a stable isotope mass spectrometer, giving a value for fraction of modern carbon.

### AMS measurement at the IAA

Pretreatment: (1) Rootlets and granules were removed using tweezers. (2) The acid–alkali–acid (AAA) pretreatment process was used for eliminating carbonates and secondary organic acids. After the treatment, the sample was neutralized with ultrapure water, and dried. In the acid treatments of the AAA, the sample is treated with HCl (1 M). In the standard alkaline treatment, the sample is treated with NaOH, by gradually raising the concentration level from 0.001 to 1 M. If the alkaline concentration reaches 1 M during the treatment, the treatment is described as AAA in the table, while AAA if the concentration does not reach 1 M. (3) The sample was oxidized by heating to produce CO_2_ gas. (4) The produced CO_2_ gas was purified in a vacuum line. (5) The purified CO_2_ gas sample was reduced to graphite by hydrogen using iron as a catalyst. (6) The produced graphite was pressed into a target holder with a hole of 1 mm diameter for the AMS ^14^C dating, using a hand-press machine.

Measurement: The graphite sample was measured against a standard of Oxalic acid (HOxII) provided by the National Institute of Standards and Technology (USA), using a ^14^C-AMS system based on the tandem accelerator. A blank for the background check was also measured.

### Data availability

The data that support the findings of this study are available from the corresponding author upon request.

## Electronic supplementary material

Supplementary Information
