# Supplementary material for: A rapid cosmic-ray increase in BC 3372–3371 from ancient buried tree rings in China
Source: Nat Commun. 2017 Nov 14;8:1487. doi: 10.1038/s41467-017-01698-8 (PMC5684315; doi:10.1038/s41467-017-01698-8)
Supplement: Supplementary file 1 — Supplementary Information [file 41467_2017_1698_MOESM1_ESM.pdf]

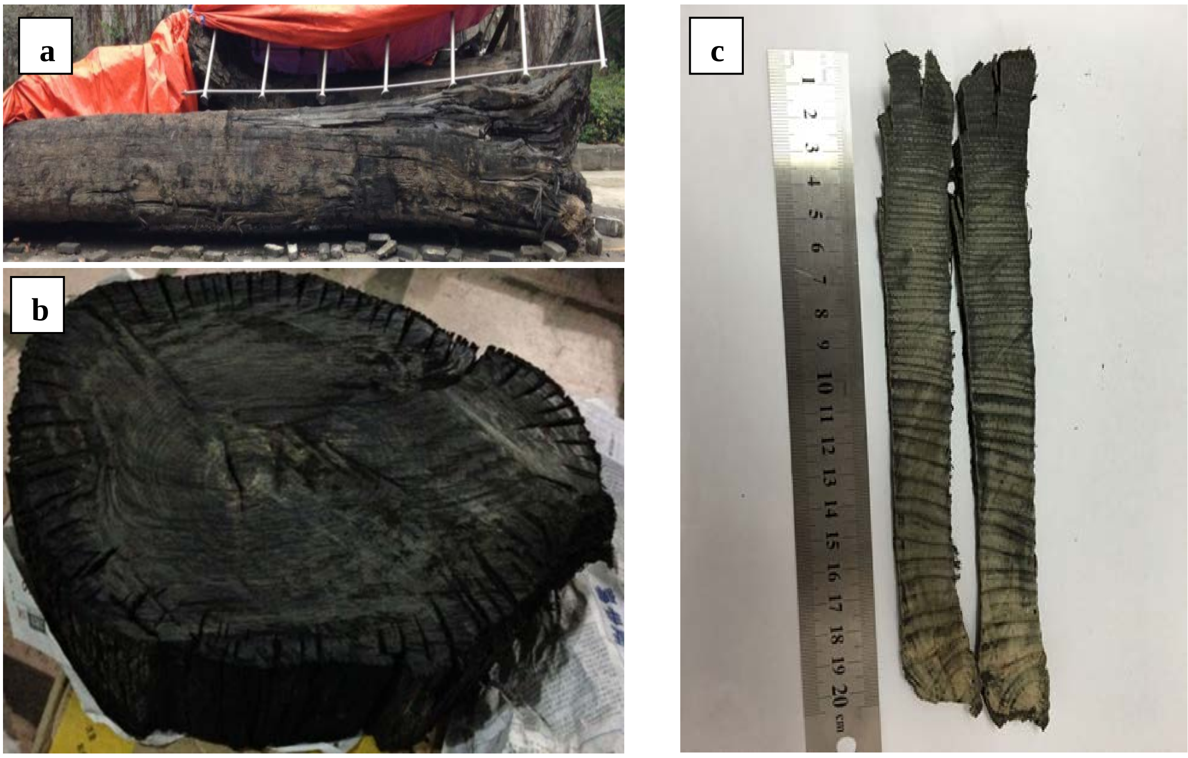

Supplementary Figure 1:The buried tree used in our analysis. *Panel a*: the buried tree in Yichang Museum, China. *Panel b*: a piece of tree. *Panel c*: tree rings cut from panel b.
